# Inter‐laboratory analytical improvement of succinylacetone and nitisinone quantification from dried blood spot samples

**DOI:** 10.1002/jmd2.12112

**Published:** 2020-04-04

**Authors:** Hilde Laeremans, Charles Turner, Tommy Andersson, Jose Angel Cocho de Juan, Adam Gerrard, M. Rebecca Heiner‐Fokkema, Diran Herebian, Nils Janzen, Giancarlo la Marca, Mattias Rudebeck

**Affiliations:** ^1^ Laboratoire de Pédiatrie ULB Brussels Belgium; ^2^ WellChild Laboratory Evelina London Children's Hospital London UK; ^3^ Sobi Stockholm Sweden; ^4^ Laboratorio de Metabolopatias Hospital Clínico Universitario de Santiago Santiago de Compostela Spain; ^5^ Newborn Screening and Biochemical Genetics Birmingham Children's Hospital Birmingham UK; ^6^ Laboratory of Metabolic Diseases University Medical Center Groningen, University of Groningen Groningen The Netherlands; ^7^ Department of General Pediatrics, Neonatology and Pediatric Cardiology, Medical Faculty Heinrich‐Heine‐University Düsseldorf Düsseldorf Germany; ^8^ Screening‐Labor Hannover Hannover Germany; ^9^ Department of Clinical Chemistry Hannover Medical School Hannover Germany; ^10^ Newborn Screening, Clinical Chemistry and Pharmacology Lab Meyer Children's University Hospital Florence Italy

**Keywords:** dried blood spot, hereditary tyrosinemia type 1, nitisinone, succinylacetone, tandem mass spectrometry

## Abstract

**Background:**

Nitisinone is used to treat hereditary tyrosinemia type 1 (HT‐1) by preventing accumulation of toxic metabolites, including succinylacetone (SA). Accurate quantification of SA during newborn screening is essential, as is quantification of both SA and nitisinone for disease monitoring and optimization of treatment. Analysis of dried blood spots (DBS) rather than plasma samples is a convenient method, but interlaboratory differences and comparability of DBS to serum/plasma may be issues to consider.

**Methods:**

Eight laboratories with experience in newborn screening and/or monitoring of patients with HT‐1 across Europe participated in this study to assess variability and improve SA and nitisinone concentration measurements from DBS by liquid chromatography‐tandem mass spectrometry (LC‐MS/MS). Quantification of nitisinone from both DBS and plasma was performed to assess sample comparability. In addition, efforts to harmonize laboratory procedures of SA and nitisinone quantifications during 5 rounds of analysis are described.

**Results:**

Nitisinone levels measured from DBS and plasma strongly correlated (*R* ^2^ = 0.93). Due to partitioning of nitisinone to the plasma, levels were higher in plasma by a factor of 2.34. In the initial assessment of laboratory performance, all had linear calibrations of SA and nitisinone although there was large inter‐laboratory variability in actual concentration measurements. Subsequent analytical rounds demonstrated markedly improved spread and precision over previous rounds, an outcome confirmed in a final re‐test round.

**Conclusion:**

The study provides guidance for the determination of nitisinone and SA from DBS and the interpretation of results in the clinic. Inter‐laboratory analytical harmonization was demonstrated through calibration improvements.

1

**SYNOPSIS**

Improved precision of succinylacetone and nitisinone quantifications from dried blood spot samples through external quality control and protocol optimization across different laboratories in Europe.

**Abbreviations**
DBS: dried blood spot
FAH: fumarylacetoacetate hydrolase
HT‐1: hereditary tyrosinemia type 1
LLOQ: lower limit of quantification
NTBC: 2‐(2‐nitro‐4‐trifluoromethyl‐benzyl)‐1,3‐cyclohexanedione, nitisinone
SA: succinylacetone, LC‐MS/MS, liquid chromatography‐tandem mass spectrometry

## 2 INTRODUCTION

Hereditary tyrosinemia type 1 (HT‐1; OMIM reference 276700) is a rare autosomal recessive disorder with an incidence of one in 100 000 worldwide.^1^ The disease is caused by a defect in the final enzyme of the tyrosine breakdown pathway: fumarylacetoacetate hydrolase (FAH; EC 3.7.1.2). The lack of FAH activity results in accumulation of toxic metabolites such as succinylacetone (SA) and succinylacetoacetate causing liver damage including hepatocellular carcinoma as well as renal dysfunction, neurologic crisis, and shorter life expectancy than for healthy individuals.^2^, ^3^, ^4^

The care for patients with HT‐1 improved dramatically with the introduction of treatment with nitisinone (2‐(2‐nitro‐4‐trifluoromethyl‐benzyl)‐1,3‐cyclohexanedione [NTBC]) in the early 2000s (approved by the Food and Drug Administration in 2002 and the European Medicines Agency in 2005, Orfadin®). To date, nitisinone is still the standard of care in combination with strict dietary restrictions to minimize phenylalanine and tyrosine intake. Treatment should be started as early as possible in life and continue without interruption to improve prognosis.^5^

Newborn screening programs allow for early HT‐1 identification and disease intervention in many countries.^6^ Urine or blood SA, or its surrogate porphobilinogen synthase activity, is currently the best screening disease marker, while tyrosine is less reliable since its levels may not be consistently raised in individuals with HT‐1^7^ and individuals with other conditions or premature infants may have elevated tyrosine levels as well.^8^, ^9^ Accuracy in SA quantification is essential to avoid false positives but more importantly to assure there are no false‐negative results during newborn screening. For individuals already undergoing treatment, monitoring of detectable levels of SA is important to determine adequacy of treatment. Normal levels of SA are below 20 nmol/L in plasma,^10^ well below the lower limit of quantitation (LLOQ) for most laboratories, where any quantifiable levels of SA indicate insufficient treatment. It is therefore essential for laboratories to establish appropriate methods with low limits of quantification for optimal management of patient treatment.

While it is essential to keep SA levels as low as possible, the dose of nitisinone should not be unnecessarily high since clinical data on long‐term usage are sparse and effects of nitisinone during pregnancy are not fully known.^11^ Nitisinone has a presumed half‐life of 54 hours and is typically dosed once or twice daily at 1 mg/kg/day.^12^, ^13^, ^14^ Monitoring nitisinone levels in combination with SA thus serve as a tool facilitating disease management, dose optimization, detection of inter‐individual variability, and monitoring treatment compliance. Easy monitoring would increase the possibilities to provide the best care possible and help individuals that, for example, find treatment compliance challenging.^15^ Monitoring using dried blood spots (DBS) has advantages to address this problem since samples can be taken by the patient/caregiver at home, are less invasive, are relatively stable, can be easily transported to the laboratory via normal postal services, and many laboratories already offer home monitoring of tyrosine on DBS to confirm dietary adherence where a single assay could thereby provide reductions in laboratory costs.^16^, ^17^, ^18^, ^19^

Here, we describe a study to evaluate the current analytical performance of SA and nitisinone measurements from DBS between different laboratories involved in patient monitoring across Europe. Moreover, we describe efforts taken to harmonize laboratory measurement procedures and highlight important aspects to consider when assessing nitisinone and SA levels in the clinic. Harmonized results allow comparable information to be generated regardless of methodology or site of analysis, thus improving patient information and outcomes.^20^

## 3 METHODS

### 3.1 Study design

The initial phase of the study aimed at evaluating the current analytical performance of seven different laboratories on the determination of SA and nitisinone from DBS by assessing any variability in concentrations reported. The comparability of nitisinone levels measured from plasma samples to samples prepared as DBS was further investigated.

The second phase aimed at harmonizing results and improving the intra‐ and inter‐laboratory differences of eight (a new laboratory group which did not participate in the initial phase joined the project for SA determination only) different laboratories through analysis of the respective standards in relation to results precision.

A total of five assay rounds were performed. DBS materials enriched with predetermined SA and nitisinone concentrations were prepared and sent blinded to the participating laboratories for analysis. New test samples were distributed for each round. The laboratories submitted results of the blinded samples to the central scientist who collated the results and a review and analysis meeting was held after each round to evaluate assay improvement and advice for further improvements in the subsequent round.

### 3.2 Laboratories

Eight laboratories with experience in either newborn screening or monitoring of patients with HT‐1 or both across Europe were included in the study for harmonization of measurements: ULB, Laboratoire de Pédiatrie, Brussels, Belgium; WellChild Laboratory at Evelina London Children's Hospital, London, UK; Screening‐Labor Hannover, Hannover, Germany; Laboratory of Metabolic Diseases, University Medical Center Groningen, Groningen, The Netherlands; Clinical Chemistry and Pharmacology Lab, Meyer Children's University Hospital, Florence, Italy; Laboratorio de Metabolopatias, Hospital Clínico Universitario de Santiago, Santiago de Compostela, Spain; Department of General Pediatrics, Neonatology, and Pediatric Cardiology, University Hospital Düsseldorf, Germany; and Newborn Screening & Biochemical Genetics, Birmingham Children's Hospital, Birmingham, UK.

### 3.3 Sample preparations

Nitisinone (Sobi, Sweden) was dissolved in dimethyl sulfoxide (DMSO) to generate a 12 mM stock solution which was diluted ×100 in whole blood from which sample standards were generated. SA (4‐6‐dioxoheptanoic acid; Sigma‐Aldrich, D1415) was dissolved in PBS to generate a 2 mM stock and finally diluted ×100 in whole blood which was already enriched with nitisinone. Standard solutions in Round 4 contained 4.42, 17.7, 44.5, 88.8 μM nitisinone and 0.0, 0.36, 2.19, 4.37, 10.9 μM SA, and standard solutions in Round 5 contained 3.84, 7.69, 23.1, 57.6, 115 μM SA. To prepare the DBS, 25 μL blood sample was pipetted on the DMPK‐C cards (FTA DMPK‐C cards, Cat no WB129243, GE Healthcare) and dried at room temperature overnight. DBS samples were sent blinded to all participating laboratories by regular mail at room temperature. For analysis Round 1, 2 sets of samples differing only with regard to the anticoagulant (Li‐Heparin or ethylenediaminetetraacetic acid [EDTA]) were tested. For analysis Rounds 2 and 3, a series of unknown concentrations of SA and nitisinone were distributed. For Rounds 4 and 5, an additional set of standard samples were distributed to be used for uniform calibration in all laboratories.

### 3.4 Liquid chromatography‐tandem mass spectrometry

All analyses in all participating laboratories were performed using liquid chromatography‐tandem mass spectrometry (LC‐MS/MS). There were considerable differences in methods for both nitisinone and SA across laboratories. Only two of the seven laboratories reporting results for both analytes used the same extract for both assays. The other five used a separate blood spot punch, and a different extraction technique for each. All seven laboratories who reported results for nitisinone used reverse‐phase chromatography, with four using a stable isotope labeled internal standard, two using mesotrione and one using external standardization. Four of eight laboratories reporting SA results used reverse‐phase chromatography while the other four used flow injection; all used stable isotope labeled SA as internal standard, and five formed a derivative as part of their sample preparation. Details of the technical differences and methods between laboratories are given in Tables 1 and 2.

**Table 1:** Succinylacetone methods overview

	Lab 1	Lab 2	Lab 3	Lab 4	Lab 5	Lab 6	Lab 7	Lab 8
**Workup procedures**								
Type of paper	Perkin Elmer 226	Ahlstrom Germany GmbH	Whatman 903	Whatman 903	Whatman 903	Whatman 903	Whatman 903 Perkin Elmer 226	TFN 179 g/m^2^ Sartorius Stedim UK
Sample pretreatment	None	Pre‐extraction of AC and AA with MeOH, drying spots with air/N_2_	EDTA−/EDTA plasma	None	EDTA tubes	None	None	None
Extraction solvent	MeOH + 3% FA	AcN:H_2_O (80:20), 150 μL	AcN:H_2_O (80:20), 0.1% FA	AcN:H_2_O (80:20) + 0.001% FA	MeOH and hydrate hydrazine	AcN:H_2_O (80:20) + hydrate hydrazine	10 μmoL/L ^13^C_5_ SA + MeOH	MilliQ H_2_O + IS + Girard T hydrazine
Internal standard	^2^H_5_ SA	5.7 dioxooctanoic acid	^2^H_5_SA	NKS‐T‐1 SUAC	^13^C_4_ SA	^2^H_5_ SA	^13^C_4_SA	^13^C_4_ SA
Punch size	3 mm	3.2 mm	3 mm	3 mm	3.2 mm	3.2 mm	3.2 mm	3.2 mm
No of punches	1	1	3	1	1	1	1	1
Extraction time	4 h	45 min	30 min	40 min	30 min	25 min	3 h	Over night
Extraction temp	RT, 22 ± 2°C + mixing	RT	60°C	50°C	37°C	37°C	RT, 15‐23°C	RT, 20‐25°C
Temporary storage of samples and standards	Standard, QC (−80°C); DBS (RT) & assayed within 4 weeks	Before analysis (RT), after analysis (4°C)	−20°C	Sample (RT), IS (−20°C)	−20°C	Sample, IS (4°C)	−20°C	Sample, control and IS (−20°C), Standard −80°C)
Maximum storage time of standard	1 year (−80°C)	1 year	1 year	3 months	1 month	3 months	1 year	2 years
**LC‐** **parameters**								
Second derivatization	None	None	Butanol + HCl	None	None	Butanol + HCl	None	None
LC column	Hichrom ACE, change to agilent Poroshell 120 EC‐C18	None, direct infusion	Phenomenex Gemini NX‐C18	None	Agilent Poroshell 120 EC‐C18	None	None, direct infusion	Phenomenex Kinetex Biphenyl
LC flow rate	0.25 mL/min	0.06 mL/min	0.3 mL/min	0.2 mL/min	0.4 mL/min	0.06 mL/min	0.02 mL/min	0.8 mL/min
LC mobile phase	Gradient, AcN:H_2_O (70:30) + 0.025% FA to AcN:H_2_O (98:2) + 0.025% FA	Isocratic, AcN:H_2_O (80:20)	Isocratic, MeCN:H_2_O (70:30) + 0.1% FA + 0.01% TFA	Isocratic, AcN:H_2_O (80:20) + 0.05% FA	Isocratic, AcN:H_2_O (85:15) + 0.05% FA	Isocratic, AcN:H_2_O (70:30) + 0.05% FA	Isocratic, MeOH:H_2_O (80:20) + 0.05% FA	Isocratic, MeOH:H_2_O + 0.1% FA (60:40)
LC temp	22 ± 2°C	RT	40°C	40°C	RT	RT	RT (15‐23°C)	RT (20‐25°C)
LC injection vol.	3 μL	20 μL	20 μL	5 μL	1 μL	40 μL	20 μL	1 μL
**Detector**								
Detector type	AB Sciex API 6500 Qtrap	Waters Xevo TQD	Waters TQD	AB Sciex API 4000	AB Sciex API 4000	AB Sciex API 4000	Waters Xevo TQD	AB Sciex API 3000 (Rounds 1–3) API 4500 (Rounds 4‐5)
Detector +/− detection	+/− switching, SA‐	ESI+	ESI+	ESI+	ESI+	ESI+	ESI+	ESI+
Detector trace transition	SA: m/z 157 > 114 (also 157 > 99); IS: m/z 157 > 118 (also 157 > 101)	SA: m/z 155 > 137; IS: m/z 169 > 151	SA: m/z 211 > 137; IS: m/z 216 > 142	SA: m/z 155 > 137; IS: m/z 160 > 142	SA: m/z 155 > 137 IS: m/z 159 > 141	SA: m/z 211 > 137 IS: m/z 216 > 142	SA: m/z 272 > 185 IS: m/z 277 > 190	R (1–5) SA: m/z 272 > 185; R (1‐5) IS: m/z 276 > 189
LLOQ	0.3 μmoL/L	1.4 μmoL/L	0.5 μmoL/L	0.5 μmoL/L	0.2 μmoL/L	0.5 μmoL/L	1.2 μmoL/L	0.55 μmoL/L
LLOD	0.15 μmoL/L	0.47 μmoL/L	0.2 μmoL/L	0.5 μmoL/L	0.1 μmoL/L	0.5 μmoL/L	0.6 μmoL/L	0.17 μmoL/L
**Reporting**								
Reporting calculation	Standard curve: liquid calibrators 0.5, 2.5, 10 μmoL/L	Liquid calibrators 0, 2, 5, 10, 20, 50 μmoL/L	Standard curve: linear, weighting 1/×	Standard curve	Standard curve + IS	Standard curve + IS	Standard curve + IS	Standard curve: blood spot calibrators 0.1, 0.25, 0.5, 1, 2.5, 5, 10, 25 μmoL/L
Cut‐off values	0.3 μmoL/L	0.3 μmoL/L (newborn screening: 2 μmoL/L)	<0.5 μmoL/L	1 μmoL/L	<0.2 μmoL/L	<1.25 μmoL/L	<1.2 μmoL/L	<0.6 μmoL/L

Abbreviations: EDTA, ethylenediaminetetraacetic acid; NTBC, 2‐(2‐nitro‐4‐trifluoromethyl‐benzyl)‐1,3‐cyclohexanedione, nitisinone; SA, succinylacetone; AA, aminoacids; AC, acylcarnitines; AcN, acetonitrile; LLOQ, lower limit of quantitation; LLOD, lower limit of detection; RT, room temperature.

**Table 2:** Nitisinone methods overview

	Lab 1	Lab 2	Lab 3	Lab 4	Lab 5	Lab 6	Lab 8
**Workup procedures**							
Type of paper	Perkin Elmer 226	Ahlstrom Germany GmbH	Whatman 903	Whatman 903	Whatman 903	Whatman 903	TFN 179 g/m^2^ Sartorius Stedim UK
Sample pretreatment	None	None	EDTA−/EDTA plasma	None	EDTA tubes	None	None
Extraction solvent	MeOH +3% FA	MeOH + IS	MeOH +0.05% FA	MeOH	MeOH & hydrate hydrazine	AcN:H_2_O (80:20) + hydrazine & IS	MeOH
Internal standard	^13^C_6_ nitisinone AlsaChim France	^13^C_6_ nitisonone	^2^H_4_ nitisinone	Mesotrione	External calibration	^13^C_6_ nitisinone Toronto Canada	Mesotrione
Punch size	3 mm	3.2 mm	3 mm	3 mm	3.2 mm	3.2 mm	3.2 mm
No of punches	1	1	3	1	1	1	1
Extraction time	4 h	30 min	30 min	40 min	30 min	25 min	30 min vortexing
Extraction temp	RT, 22 ± 2°C	RT	RT	RT	37°C	37°C	RT (20–25°C)
Temporary storage of samples and standards	Standard, QC (−80°C); DBS (RT) & assayed within 4 weeks	Calibrator, control, serum sample (−20°C), sample (4°C)	−20°C	Sample (RT), standard (−20°C)	−20°C	4°C	Sample, control, IS (−20°C), standards (−80°C)
Maximum storage time of standard	1 year (−80°C)	1 year	1 year	3 months	1 month	3 months	2 years
**LC‐** **parameters**							
LC column	Hichrom ACE, change to Agilent Poroshell 120 EC‐C18	UPLC BEH C18 Waters	Phenomenex Gemini NX‐C18	Phenomenex Gemini NX‐C18	Agilent Poroshell 120 EC‐C18	Agilent Poroshell 120 EC‐C18	Phenomenex Luna NH2
LC flow rate	0.25 mL/min	0.45 mL/min	0.3 mL/min	0.2 mL/min	0.4 mL/min	0.5 mL/min	0.4 mL/min
LC mobile phase	Gradient AcN:H_2_O (70:30) + 0.025% FA to AcN:H_2_O (98:2) + 0.025% FA	Isocratic, AcN:H_2_O (50:50) + 0.1% FA + 0.01% TFA	Isocratic, AcN:H_2_O (70:30) + 0.1% FA + 0.01% TFA	Isocratic, AcN:H_2_O (60:40) + 0.1% FA + 0.01% TFA	Isocratic, AcN:H_2_O (85:15) + 0.05% FA	Isocratic, AcN:H_2_O (85:15) + 0.05% FA	Isocratic AcN:H_2_O (90:10) + 0.1% FA
LC temp	22 ± 2°C	40°C	40°C	40°C	RT	30°C	RT (20‐25°C)
LC injection vol.	3 μL	7.5 μL	10 μL	5 μL	1 μL	10 μL	1 μL
Detector							
Detector type	AB Sciex API 6500 Qtrap	Waters Xevo TQ MS	Waters TQD	AB Sciex API 4000	AB Sciex API 4000	AB Sciex API 4000	AB Sciex API 3000 (Rounds 1–3) API 4500 (Rounds 4‐5)
Detector +/− detection	+/− switching, NTBC+	ESI+	ESI+	ESI+	ESI+	ESI+	ESI+
Detector trace transition	NTBC: m/z 330 > 218 (also 330 > 126); IS: m/z 336 > 218 (also 336 > 126)	NTBC: m/z 330 > 218; IS: m/z 336 > 218	NTBC: m/z 330 > 218; IS: m/z 334 > 218	NTBC: m/z 330 > 218 (also 330 > 126); IS: m/z 340 > 228 (also 340 > 104)	NTBC: m/z 330 > 218 (also 330 > 126)	NTBC: m/z 330 > 218 (also 330 > 126) IS: m/z 336 > 218 (also 336 > 126)	NTBC: m/z 330 > 218; IS: m/z 340 > 228
LLOQ	0.5 μmoL/L	0.08 μmoL/L	0.5 μmol/L	0.5 μmoL/L	0.25 μmoL/L	0.5 μmoL/L	2.2 μmoL/L
**Reporting**							
Reporting calculation	Standard curve: liquid calibrators 5, 10, 50, 100 μmoL/L	Liquid calibrators 0, 0.1, 1, 5, 20, 40, 60, 100 μmoL/L	Standard curve: linear, weighting 1/×	Standard curve	Standard curve	Standard curve + IS	Standard curve: Blood spot calibrators 1, 2.5, 5, 10, 25, 50, 100, 150 μmoL/L

Abbreviations: DBS, dried blood spots; EDTA, ethylenediaminetetraacetic acid; NTBC, 2‐(2‐nitro‐4‐trifluoromethyl‐benzyl)‐1,3‐cyclohexanedione, nitisinone; SA, succinylacetone; AA, aminoacids; AC, acylcarnitines; AcN, acetonitrile; LLOQ, lower limit of quantitation; LLOD, lower limit of detection; RT, room temperature.

### 3.5 Statistics

Linear regression analysis was performed to investigate the correlation between nitisinone calculated from DBS vs plasma and samples prepared with EDTA vs Lithium‐Heparin. A two‐sided *t*‐test was used to evaluate the impact of the anticoagulant on the results. Expected vs measured SA and nitisinone concentrations were plotted and trendlines calculated.

## 4 RESULTS

### 4.1 Quantification of nitisinone from DBS and plasma

**Figure 1:**
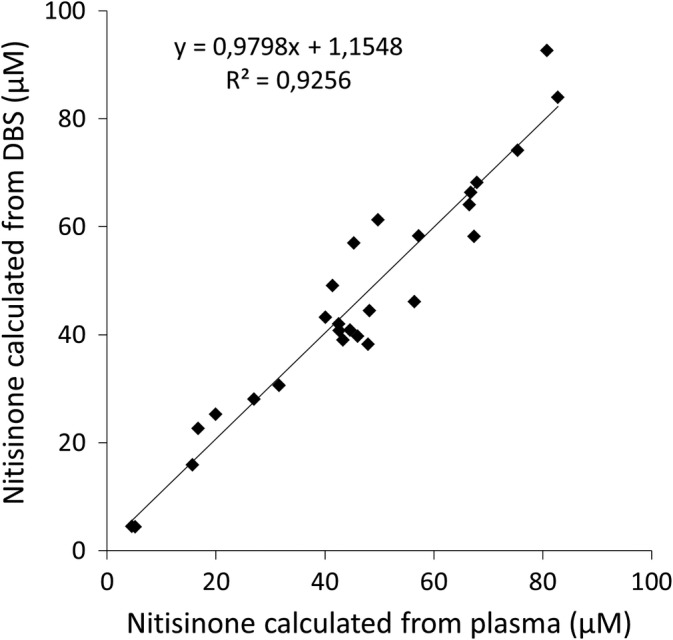
Quantification of nitisinone from dried blood spots (DBS) and plasma. Samples from patients treated with nitisinone analysed from plasma and from DBS. Each dot represents data from one patient (N = 27). DBS values multiplied by conversion factor of 2.34

Nitisinone measurement in plasma by LC‐MS/MS is well established.^21^ To test if nitisinone levels measured from plasma samples were comparable to samples prepared as DBS, concentrations in 27 blood samples were analysed by LC‐MS/MS at two different laboratories in the study (Figure 1). The concentrations significantly correlated between samples (*R* ^2^ = 0.9256) indicating that either way of sample preparation could be used during clinical assessment of patient samples. The reported conversion factors between DBS values vs plasma values vary. A conversion factor of 2.4 in 9 paired plasma and DBS samples was found by one research group,^21^ while a second group derived a factor of 2.6 from 39 paired samples from 13 patients^22^ (poster abstract). Our own data indicate a conversion factor of 2.34.

### 4.2 Initial assessment of SA and nitisinone quantifications

**Figure 2:**
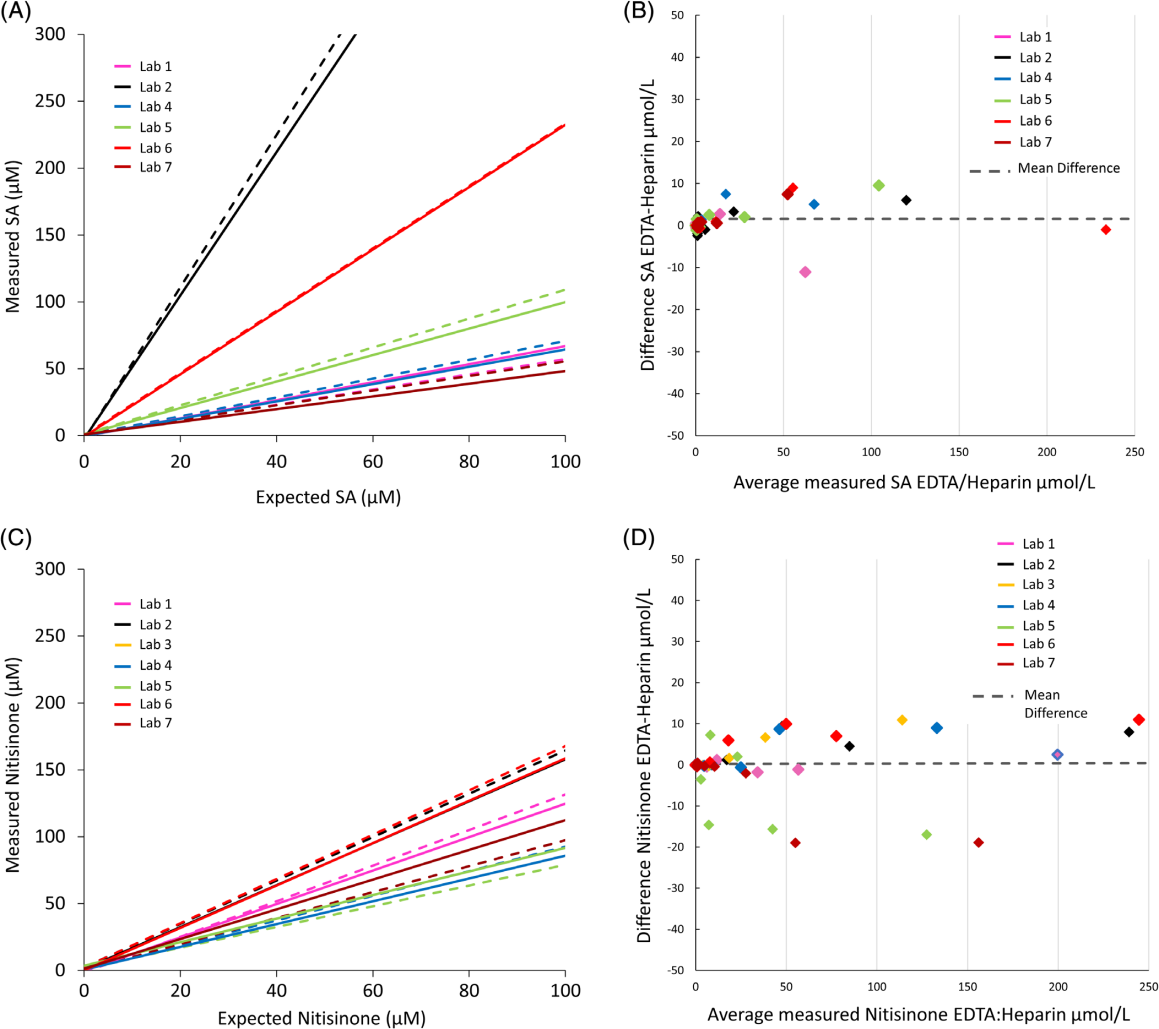
A, Assessment of succinylacetone (SA) concentration from dried blood spots (DBS). Blood samples spiked with 0.0, 0.3, 1.0, 5.0, 25.0, 100.0 μM SA were assessed by six laboratories. Trend lines were drawn to illustrate linear relationship (all *R* ^2^ values >0.99). Dotted lines represent results from tubes using ethylenediaminetetraacetic acid (EDTA) as anticoagulant and solid lines represent results from tubes using Li‐Heparin. Outliers not shown in figure: Laboratory 2 had values of 571.0 (EDTA) and 538.0 (Li‐Heparin) for the 100.0 μM sample. B, Difference plot comparing succinylacetone (SA) results from DBS prepared from blood tubes using EDTA as anticoagulant and those from tubes using Li‐Heparin showing no significant difference (*P* > .05). C, Assessment of nitisinone concentration from DBS. Blood samples spiked with 0.0, 5.0, 10.0, 25.0, 50.0, 150.0 μM nitisinone were assessed by seven laboratories. Trend lines were drawn to illustrate linear relationship (all *R* ^2^ values >0.97). Dotted lines represent results from tubes using EDTA as anticoagulant and solid lines represent results from tubes using Li‐Heparin. D, Difference plot comparing nitisinone results from DBS prepared from blood tubes using EDTA as anticoagulant and those from tubes using Li‐Heparin showing no significant difference (*P* > .05)

Seven clinical laboratories were included in an initial evaluation to test if measured SA and nitisinone concentrations vary between laboratories to a clinically significant extent, which could motivate harmonization of laboratory measurement procedures. In the first assessment round, all laboratories received 2 × 8 blinded DBS samples containing both SA and nitisinone, prepared either from tubes with EDTA or Li‐Heparin as anticoagulant. SA and nitisinone were quantified by LC‐MS/MS according to the standard procedure in each laboratory. Six laboratories returned results for the SA determination, and all laboratories for nitisinone. There was a highly linear relationship between the measured and the expected concentration of both SA and nitisinone (*R* ^2^ > 0.9798 for each laboratory). However, most laboratories either over‐ or underestimated the concentrations to an extent (−23% to +65% for nitisinone, and − 45% to +570% for SA) which could have a clinical impact with relation to dosing of nitisinone, particularly for SA (Figure 2A,B). The use of either Li‐Heparin or EDTA as anticoagulant did not impact the results (Figure 2C,D).

**Figure 3:**
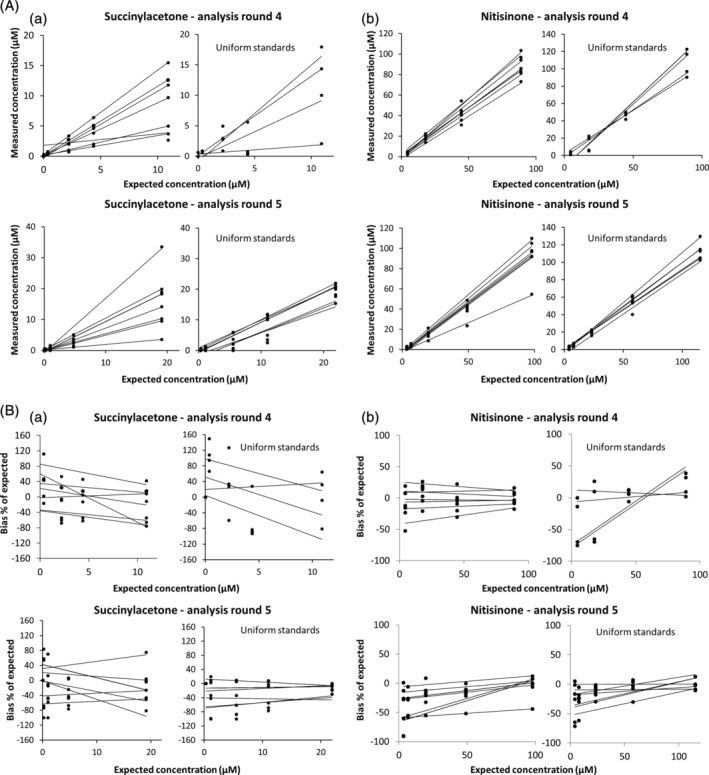
Outcomes of analysis Rounds 4 and 5. A, Measured succinylacetone (SA) and nitisinone concentrations plotted against the expected concentrations demonstrate markedly improved spread and precision. B, Bias plots of percentage deviation from target values of measured SA and nitisinone concentrations demonstrate improved accuracy and precision in Round 5

Several of the laboratories had incorrect assessment of the lower concentrations of SA or of samples absent of added SA (Figure S1A,B), motivating technical improvements in preparations for the following analysis round. Most problems were due to extraction difficulties and calibration differences. Thus, taken together the first assessment round showed a need for improving laboratory performance for quantification of both nitisinone and SA.

### 4.3 Improving analytical quality of SA and nitisinone quantification

The agreement between laboratories in assessing SA and nitisinone improved in the following analysis round: the quality in Round 2 was considered good but there were still discrepancies attributable to calibration (between laboratory slope bias against added amount). This persisted in Round 3. It was also considered by the study group that some of the added levels both of SA and nitisinone were too high and not reflecting concentrations found clinically (Figure S1A,B).

For analysis Round 4, samples with lower concentrations of both SA and nitisinone were distributed to test assay performance in a concentration range that was clinically more relevant than that tested in previous rounds. In addition, new liquid standards were included to test if calibration with the same samples would improve assay performance. In this round, the spread and precision markedly improved over the previous rounds (Figure 3A,B).

In Round 5, the standardization was retested with the same type of samples and preparations of Round 4 and the improvement seen in Round 4 was confirmed (Figure 3A,B).

A summary of which laboratory participated in which analysis round is presented in Table S1.

## 5 DISCUSSION

From the observed results of this study, one of our overarching goals is to provide guidance and highlight pitfalls for other clinical laboratories and researchers about the quantification of SA and nitisinone specifically on DBS. DBS is an attractive alternative to plasma to use for the clinical follow‐up of patients. It offers the patients the opportunity to perform the sampling themselves without loss of quality and clinical value of the results for the physician and the patients.

However, quantification of different substances in DBS is challenging and many physicians doubt the accuracy of the results from a DBS. This doubt can be the consequence of interlaboratory differences of reported values and lack of knowledge about the matrix and methods. It is therefore important to overcome the differences between laboratories. These differences are the consequence of poor recovery of the substances, poor limits of quantification, calibration issues or calculation errors. Therefore, we strive to harmonize the existing protocols for SA and nitisinone to that level that results between laboratories are comparable.

All contributing laboratories shared their protocols, instruments, and chemicals used, in order to start the evaluation. In the first step, all laboratories used their daily used methods without any modifications. However, it should be stated that some of the laboratories were just starting with these methods and were not offering a day‐to‐day clinical service. This first round of data, together with the comparison of the methods, led to a first modification of local protocols as well as a harmonization across protocols. Furthermore, we excluded some of the potential interferences like the differences in the type and use of blood tubes. The results clearly indicated no significant influence of the anticoagulant of the blood tubes.

A second step in order to come to a harmonized protocol was to use the same calibration standards. An independent industrial partner provided external quality control samples but also standard curves. Use of common calibration material further reduced the slight differences between the laboratories, indicating the importance of correct selection of standard curves with the means available.

In the process of method comparisons, laboratories referred to the calibrator supplied with the device and thus achieved a significantly better agreement in the given concentrations, however, specific modifications to the methods contributed to the improvement in reported analytical values. These modifications were related to the extraction temperature where individual laboratories modified their method from 40°C to 60°C for SA and 40°C to room temperature for nitisinone. LC temperature was modified by individual laboratories from room temperature to 40°C while the extraction time for SA was changed from 45 to 30 minutes. In addition, a change in LC‐column for nitisinone was made by a laboratory from Luna C18 to Gemini NX‐C18.

As described above, correct understanding of the meaning of the reported values of these two markers by physicians is crucial. Even after initiating nitisinone treatment, SA concentrations in urine decrease rapidly and are usually not detectable after 1 to 2 days. But in blood SA concentrations decrease more slowly. The half‐life of SA is approximately 12 days (unpublished calculations from the data of the initial registration study for nitisinone “the NTBC study”). It takes well over a month before SA in blood becomes unquantifiable and therefore within normal limits. For nitisinone on the other hand, the half‐life is approximately 2 days (1 day in newborns).^23^, ^24^ A steady‐state concentration is obtained after 12 days dosing.^24^

A limitation of the study is that time/stability and temperature stability studies were not performed, and data on time of analysis at each laboratory from preparation of samples were not collected for the calculation of recovery rates. However, only about one‐third of the increased concentrations of SA is measured from DBS. The recovery rate for SA of about 30% is thereby much lower than for nitisinone which is about 95%.

A DBS external quality control scheme including nitisinone and SA is now available from an independent provider. However, further work is still needed in the future to harmonize the quantification of nitisinone and SA with LC‐MS/MS, preferably by performing a study enabling data providing scientific evidence for development of a standard protocol.

In conclusion, this article provides guidance for the determination of nitisinone and SA from DBS and the interpretation of results in the clinic. Furthermore, inter‐laboratory analytical improvements were demonstrated through calibration improvements. This should be minimized through the use of common reference samples across laboratories. DBS show to be a good matrix for regular clinical follow‐up of patients with a minimum of burden for the patients and optimal clinical surveillance and guidance.

## CONFLICT OF INTEREST

C.T. received grants from Sobi, and is a Director of SpotOn Clinical Diagnostics, a spinout company of Guy's and St Thomas NHS Foundation Trust and King's College London. N.J. is owner and head of the screening laboratory Hannover. T.A. and M.R. are employees and shareholders of Sobi. All other authors declare no potential conflict of interest. This study was fully funded by Sobi.

## AUTHOR CONTRIBUTIONS

H.L., C.T., J.A.C.J., A.G., M.R.H.‐F., D.H., N.J., and G.M. planned and designed the study, performed the analysis, evaluated study results, participated in drafting and revising of the manuscript and read and approved the final version before submission. T.A. planned and designed the study, central scientist, evaluated study results, participated in drafting and revising of the manuscript and read and approved the final version before submission. M.R. planned and designed the study, project director, evaluated study results, participated in drafting and revising of the manuscript and read and approved the final version before submission.

## Supporting information


**Figure S1A** *Outcomes of analysis Rounds 1*, *2 and 3*. Initial evaluation of inter‐laboratory variability demonstrated a need for technical improvements and harmonization. Rounds 2 and 3 showed improved intra‐laboratory performance and agreement between laboratories with persisting discrepancies attributed to calibration motivating further analysis rounds.

**Figure S1B** *Outcomes of analysis Rounds 1*, *2 and 3*. Bias plots of percentage deviation from target values of measured succinylacetone and nitisinone concentrations.

**Table S1** Laboratory participation per analysis round

## References

[1] Hutchesson AC , Hall SK , Preece MA , Green A . Screening for tyrosinaemia type I. Arch Dis Child Fetal Neonatal Ed. 1996;74(3):F191‐F194. 8777683 10.1136/fn.74.3.f191 PMC2528336

[2] Mitchell GA et al. *Hypertyrosinemia* In: Scriver CR et al., eds. The Metabolic and Molecular Basis of Inherited Disease. New York: McGraw‐Hill Medical; 2001:1777‐1805.

[3] Lindblad B , Lindstedt S , Steen G . On the enzymic defects in hereditary tyrosinemia. Proc Natl Acad Sci U S A. 1977;74(10):4641‐4645. 270706 10.1073/pnas.74.10.4641 PMC432003

[4] van Ginkel WG , Jahja R , Huijbregts SCJ , et al. Neurocognitive outcome in tyrosinemia type 1 patients compared to healthy controls. Orphanet J Rare Dis. 2016;11(1):87. 27356512 10.1186/s13023-016-0472-5 PMC4928338

[5] de Laet C , Dionisi‐Vici C , Leonard JV , et al. Recommendations for the management of tyrosinaemia type 1. Orphanet J Rare Dis. 2013;8:8. 23311542 10.1186/1750-1172-8-8 PMC3558375

[6] Giguere Y , Berthier MT . Newborn screening for hereditary Tyrosinemia type I in Quebec: update. Adv Exp Med Biol. 2017;959:139‐146. 28755192 10.1007/978-3-319-55780-9_13

[7] Goulden KJ , Moss MA , Cole DEC , Tithecott GA , Crocker JFS . Pitfalls in the initial diagnosis of tyrosinemia: three case reports and a review of the literature. Clin Biochem. 1987;20(3):207‐212. 3308177 10.1016/s0009-9120(87)80122-4

[8] Morrissey MA , Sunny S , Fahim A , Lubowski C , Caggana M . Newborn screening for Tyr‐I: two years’ experience of the New York state program. Mol Genet Metab. 2011;103(2):191‐192. 21441051 10.1016/j.ymgme.2011.02.017

[9] Wilcken B , Haas M , Joy P , et al. Expanded newborn screening: outcome in screened and unscreened patients at age 6 years. Pediatrics. 2009;124(2):e241‐e248. 19620191 10.1542/peds.2008-0586

[10] Cyr D , Giguère R , Villain G , Lemieux B , Drouin R . A GC/MS validated method for the nanomolar range determination of succinylacetone in amniotic fluid and plasma: an analytical tool for tyrosinemia type I. J Chromatogr B Analyt Technol Biomed Life Sci. 2006;832(1):24‐29. 10.1016/j.jchromb.2005.12.007 16414314

[11] Barchanska H , Rola R , Szczepankiewicz W , Mrachacz M . LC‐MS/MS study of the degradation processes of nitisinone and its by‐products. J Pharm Biomed Anal. 2019;171:15‐21. 30959315 10.1016/j.jpba.2019.03.046

[12] (EMA), E.M.A., *Summary of Product Charateristics: Orfadin®* Available at: http://www.ema.europa.eu/docs/en_GB/document_library/EPAR_-_Product_Information/human/000555/WC500049195.pdf (Accessed in April 2018)

[13] Guffon N , Bröijersén A , Palmgren I , Rudebeck M , Olsson B . Open‐label single‐sequence crossover study evaluating pharmacokinetics, efficacy, and safety of once‐daily dosing of Nitisinone in patients with hereditary Tyrosinemia type 1. JIMD Rep. 2018;38:81‐88. 28643275 10.1007/8904_2017_29 PMC5874213

[14] Mayorandan S , Meyer U , Gokcay G , et al. Cross‐sectional study of 168 patients with hepatorenal tyrosinaemia and implications for clinical practice. Orphanet J Rare Dis. 2014;9:107. 25081276 10.1186/s13023-014-0107-7 PMC4347563

[15] Couce ML , Dalmau J , del Toro M , Pintos‐Morell G , Aldámiz‐Echevarría L , Spanish Working Group on Tyrosinemia type1 . Tyrosinemia type 1 in Spain: mutational analysis, treatment and long‐term outcome. Pediatr Int. 2011;53(6):985‐989. 21752152 10.1111/j.1442-200X.2011.03427.x

[16] Wagner M , Tonoli D , Varesio E , Hopfgartner G . The use of mass spectrometry to analyze dried blood spots. Mass Spectrom Rev. 2016;35(3):361‐438. 25252132 10.1002/mas.21441

[17] la Marca G , Malvagia S , Materazzi S , et al. LC‐MS/MS method for simultaneous determination on a dried blood spot of multiple analytes relevant for treatment monitoring in patients with tyrosinemia type I. Anal Chem. 2012;84(2):1184‐1188. 22148291 10.1021/ac202695h

[18] Sander J , Janzen N , Terhardt M , et al. Monitoring tyrosinaemia type I: blood spot test for nitisinone (NTBC). Clin Chim Acta. 2011;412(1–2):134‐138. 20883679 10.1016/j.cca.2010.09.027

[19] Zakaria R et al. Advantages and challenges of dried blood spot analysis by mass spectrometry across the Total testing process. Ejifcc. 2016;27(4):288‐317. 28149263 PMC5282914

[20] Plebani M . Harmonization of clinical laboratory information ‐ current and future strategies. EJIFCC. 2016;27(1):15‐22. 27683502 PMC4975213

[21] Prieto JA , Andrade F , Lage S , Aldámiz‐Echevarría L . Comparison of plasma and dry blood spots as samples for the determination of nitisinone (NTBC) by high‐performance liquid chromatography‐tandem mass spectrometry. Study of the stability of the samples at different temperatures. J Chromatogr B Analyt Technol Biomed Life Sci. 2011;879(11–12):671‐676. 10.1016/j.jchromb.2011.01.031 21377430

[22] Fuenzalida K et al. *P72*. *Parallel Determination of Nitisinone Levels in Dried Blood Spot and Plasma Samples of Chilean Tyrosinemia 1 Patients by using LCMSMS*., in *Screening Pathways through China*, *the Asia Pacific Region*, *the World* . 2019;5(26):58‐59.

[23] Hall MG , Wilks MF , Provan WML , Eksborg S , Lumholtz B . Pharmacokinetics and pharmacodynamics of NTBC (2‐[2‐nitro‐4‐fluoromethylbenzoyl]‐1,3‐cyclohexanedione) and mesotrione, inhibitors of 4‐hydroxyphenyl pyruvate dioxygenase (HPPD) following a single dose to healthy male volunteers. Br J Clin Pharmacol. 2001;52(2):169‐177. 11488774 10.1046/j.0306-5251.2001.01421.x PMC2014534

[24] Huledal G , Olsson B , Önnestam K , et al. Non randomized study on the potential of nitisinone to inhibit cytochrome P450 2C9, 2D6, 2E1 and the organic anion transporters OAT1 and OAT3 in healthy volunteers. Eur J Clin Pharmacol. 2019;75(3):313‐320. 30443705 10.1007/s00228-018-2581-7

