# Supplementary figures and images for: Inter‐laboratory analytical improvement of succinylacetone and nitisinone quantification from dried blood spot samples
Source: JIMD Rep. 2020 Apr 4;53(1):90–102. doi: 10.1002/jmd2.12112 (PMC7203657; doi:10.1002/jmd2.12112)

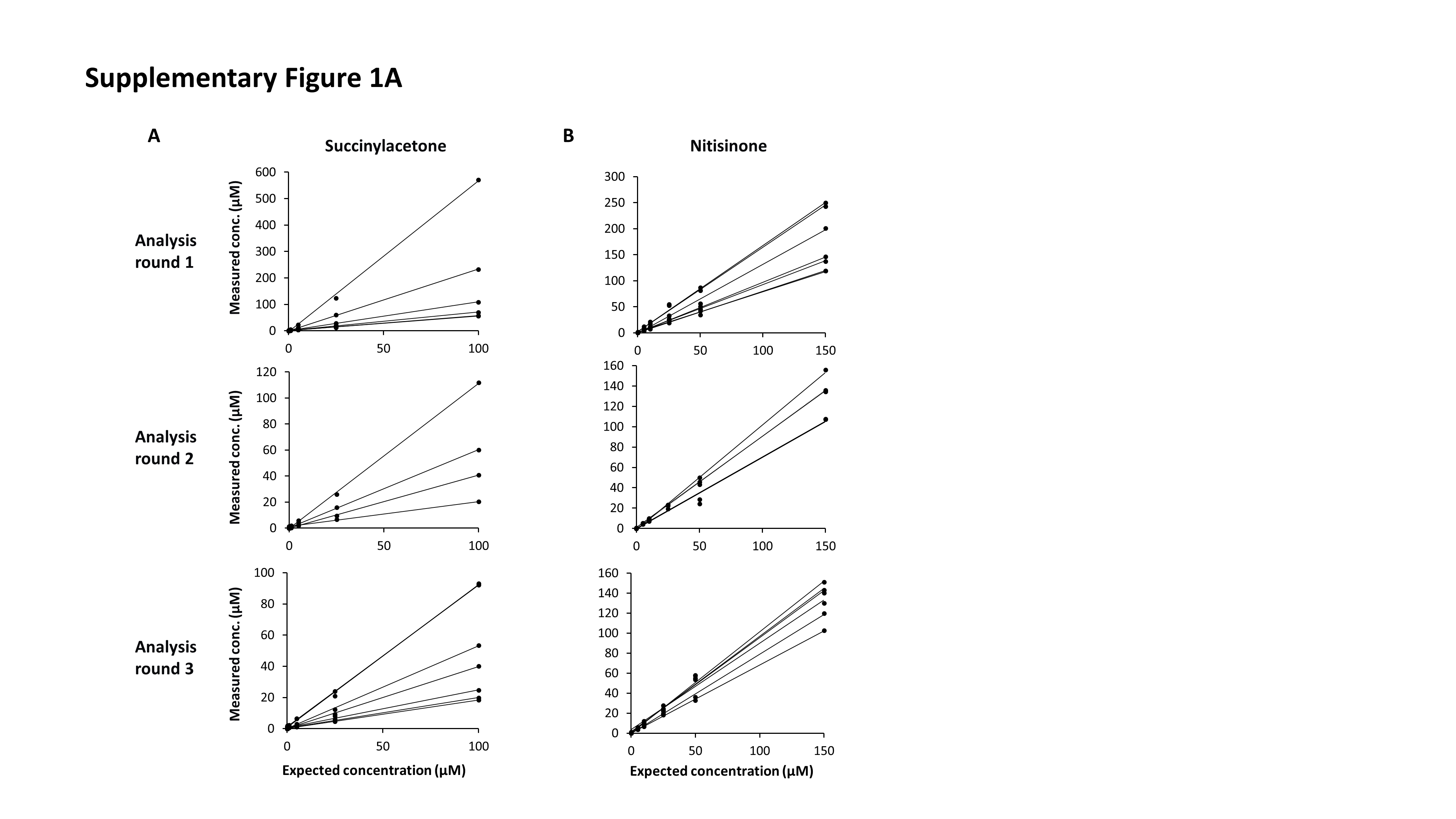

Supplement: Supplementary file 1 — Figure S1A Outcomes of analysis Rounds 1, 2 and 3. Initial evaluation of inter‐laboratory variability demonstrated a need for technical improvements and harmonization. Rounds 2 and 3 showed improved intra‐laboratory performance and agreement between laboratories with persisting discrepancies attributed to calibration motivating further analysis rounds. [file JMD2-53-90-s001.tif]

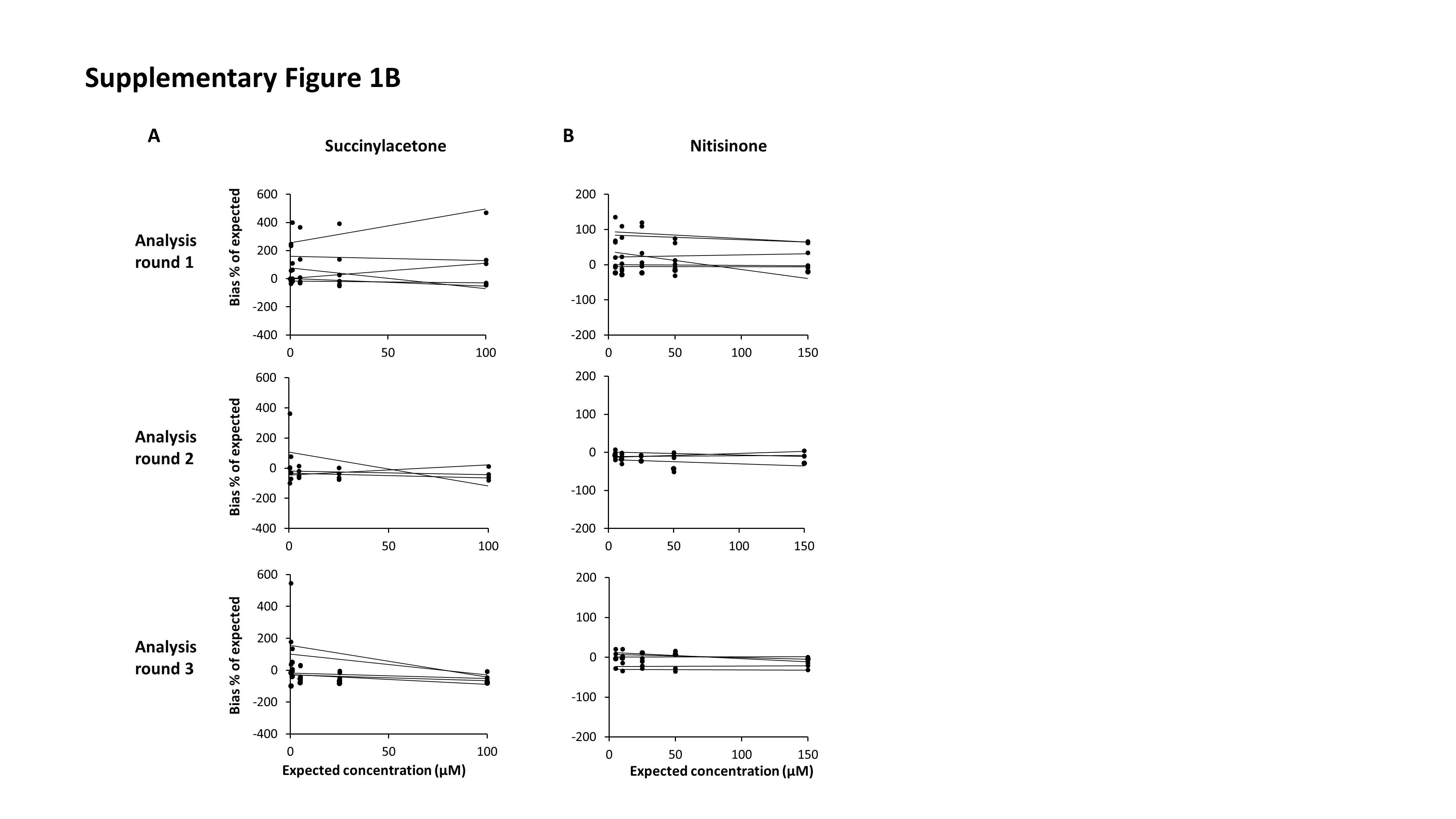

Supplement: Supplementary file 2 — Figure S1B Outcomes of analysis Rounds 1, 2 and 3. Bias plots of percentage deviation from target values of measured succinylacetone and nitisinone concentrations. [file JMD2-53-90-s003.tif]
