# Supplementary material for: Inter‐laboratory analytical improvement of succinylacetone and nitisinone quantification from dried blood spot samples
Source: JIMD Rep. 2020 Apr 4;53(1):90–102. doi: 10.1002/jmd2.12112 (PMC7203657; doi:10.1002/jmd2.12112)
Supplement: Supplementary file 3 — Table S1 Laboratory participation per analysis round [file JMD2-53-90-s002.docx]

**Supplementary Table 1.** Laboratory participation per analysis round.

| **Lab** | **Round  1** | **Round  2** | **Round  3** | **Round  4** | **Round  5** |
| --- | --- | --- | --- | --- | --- |
| **1** | X | X | X | X | X |
| **2** | X | X | X | X | X |
| **3** | X (nitisinone) | X (nitisinone) | X | X | X |
| **4** | X | X | X | X | X |
| **5** | X |  | X | X | X |
| **6** | X | X |  | X | X |
| **7** |  |  | X (SA) |  | X (SA) |
| **8** | X |  | X | X | X |
| **Total** | **7** | **5** | **7** | **7** | **8** |
